# Polymeric Microfluidic Devices Fabricated Using Epoxy Resin for Chemically Demanding and Day-Long Experiments

**DOI:** 10.3390/bios12100838

**Published:** 2022-10-07

**Authors:** Jaeseok Lee, Minseok Kim

**Affiliations:** 1 Department of Mechanical System Engineering, Kumoh National Institute of Technology, Gumi 39177, Korea; 2 Department of Aeronautics, Mechanical and Electronic Convergence Engineering, Kumoh National Institute of Technology, Gumi 39177, Korea

**Keywords:** microfluidics, soft lithography, polymeric device, epoxy resin, particle compartmentalization

## Abstract

*Polydimethylsiloxane* (PDMS) is a widely used material in laboratories for fabricating microfluidic devices with a rapid and reproducible prototypingability, owing to its inherent properties (e.g., flexibility, air permeability, and transparency). However, the PDMS channel is easily deformed under pressures applied to generate flows because of its elasticity, which can affect the robustness of experiments. In addition, air permeability of PDMS causes the pervaporation of water, and its porous structure absorbs oil and even small hydrophobic molecules, rendering it inappropriate for chemically demanding or day-long experiments. In this study, we develop a rapid and reproducible fabrication method for polymer-based rigid microfluidic devices, using epoxy resin that can overcome the limitations of PDMS channels, which are structurally and chemically robust. We first optimize a high-resolution fabrication protocol to achieve convenient and repeatable prototyping of polymeric devices via epoxy casting using PDMS soft molds. In addition, we compare the velocity changes in PDMS microchannels by tracking fluorescent particles in various flows (~133 μL/min) to demonstrate the structural robustness of the polymeric device. Furthermore, by comparing the adsorption of fluorescent hydrophobic chemicals and the pervaporation through channel walls, we demonstrate the excellent chemical resistance of the polymeric device and its suitability for day-long experiments. The rigid polymeric device can facilitate lab-on-chip research and enable various applications, such as high-performance liquid chromatography, anaerobic bacterial culture, and polymerase chain reaction, which require chemically or physically demanding experiments.

## 1. Introduction

The advantages of microfluidic devices, such as low reagent consumption [1,2], parallel analysis [3], and prevention of contamination owing to closed systems [4], render it suitable for application to various engineering fields for the purpose of providing solutions to environmental and biological problems [5,6,7,8]. In addition to the aforementioned advantages, the portability and rapid analysis afforded by microfluidic devices make them an effective diagnostic tool (e.g., for pathogen and heavy metal ion detections) [5,9,10,11]. In the early stages, microfluidic devices were fabricated using glass and silicon [12]; however, as the development of soft lithography progressed, various silicone elastomeric materials began to be used to fabricate microfluidic devices [13]. In addition, currently, various materials widely used to fabricate microchips include paper [10,11], ceramics [14,15], acrylics [16], and hydrogels [17].

Among various candidates to fabricate microfluidic devices, *polydimethylsiloxane* (PDMS) has been widely used in laboratories due to easy and rapid prototyping. PDMS-based microfluidic devices are disposable and inexpensive [18], and mechanical properties such as optical transparency, flexibility, and air permeability are the main advantages that render PDMS a suitable material for experiments [13,19]. However, PDMS channels seem to be less suitable in chemically demanding experiments requiring the use high solubility solvents (e.g., *diisopropylamine*, *triethylamine*, *pentane*, and *xylene*) or highly reactive organic reagents (e.g., *tetrabutylammonium fluoride* and *dipropylamine*) that can separate PDMS from a glass substrate or dissolve PDMS channel walls, resulting in geometry deformation [20]. In addition, since PDMS-based microchannels are prone to swelling due to absorption of solvents [21,22], uniform coating should be applied to the channels’ surfaces, using chemicals such as *Parylene-C* [23] and *poly(urethane acrylate)* [24] to prevent PDMS swelling and polymer breakdown. This requires professional bonding processes in cleanroom environments with expensive equipment and experts. With regard to rigidity, PDMS microchips are less appropriate for high-pressure operations because the PDMS channel geometry is significantly deformed, owing to low elastic modulus (*E* = 2.5 MPa) [25]. These disadvantages result in low reliability for chemically demanding and day-long experiments [26].

In order to overcome the limitations of PDMS, researchers used alternative materials that do not require surface coating, and which have strong mechanical properties. Inorganic materials (e.g., glass and silicon) can be considered suitable alternatives to PDMS due to their stable surface zeta potential [27,28,29], deformation resistance, and low air-permeability [13,30]. However, challenges encountered in fabrication processes include the management of hazardous chemicals such as *hydrofluoric acid* and glass–glass bonding processes, which typically require high temperatures (>605 °C), high pressures (>0.4 MPa), and clean environments [31,32,33]. Another problem is that the silicon substrate is opaque to visible light, making it difficult to observe the channel with an optical microscope [34].

Polymer also appears to be an alternative material to PDMS. *Cyclic olefin copolymer* (COC) has excellent resistance to polar solvents such as acids, bases and *isopropyl alcohol,* rendering it suitable for chemically demanding experiments [35]. In addition, the humidity change inside the channel is negligible due to its low water absorption (<0.01%) [36], and the high transparency of COC facilitates optical detection [37]. However, COC is easily damaged by non-polar organic solvents such as *toluene* and *naphtha* [38], and the fabrication process requires special equipment [38,39]. Another widely used polymer for microdevice fabrication, *polypropylene* (PP), is utilized for microchannel fabrication owing to its cost efficiency, high chemical resistance, and high transparency [40,41]. In particular, PP has been used in biological applications [42] due to its nontoxicity and reliability in biochemical reactions [43]. Nonetheless, because the bonding process of PP components to a substrate is sensitive to temperature, pressure, and time, the inaccurate control of these parameters may result in channel collapse and geometric changes [42].

Among polymers, epoxy resin has the potential for mass production due to its fabrication compatibility with soft lithography or photo lithography. Furthermore, epoxy resin provides high mechanical properties such as transparency and solvent resistance as shown in Table 1. For example, Raffy et al. fabricated manually resealable and reusable epoxy-based microfluidic chips at room temperature [44]. Composed of soft and stiff layers, the microfluidic device can be bonded to substrates of a variety of materials (glass, metal, and silicon), including epoxies. Cheng et al. fabricated a whole polymer-based microchip using enclosed casting of epoxy resins [45]. Due to the rigidity of the device, the width and height of the microdevice were only slightly deformed (<4%) at a pressure of 600 kPa. Sticker et al. suggested *thiol-ene* epoxy thermoset (OSTEMER) as an alternative material due to several advantages, such as its fast and easy fabrication, chemical inertness, and biocompatibility [46,47]. However, although many studies have fabricated microfluidic devices using epoxy resin, mass production and wafer-level large-area fabrication of microdevices have yet to be demonstrated.

In addition to materials, fabrication methods are also important design parameters for production of microfluidic devices. In general, a material can be used for microfluidic devices with multiple fabrication methods, allowing researchers to select a fabrication method that is appropriate for the lab environment. For example, COC microfluidic devices can be fabricated using various fabrication techniques, such as hot embossing, injection molding, and micromilling. However, hot embossing and injection molding are rarely employed in the microdevice fabrication using inorganic materials. Micromilling enables the microchannel fabrication by partially removing hard material, including inorganic materials, with a cutting tool [52,53,54]. Half an hour or less is enough for fabricating single-level simple designs [55]. Nonetheless, the surface roughness resulting from the milling process is not suitable for applications requiring optical transparency [55], and although some manufacturers provide sophisticated micromilling tools [56], the resolution is often limited to a width of 100 μm due to cost and throughput [56,57]. On the other hand, tape bonding was attempted for bonding rigid materials such as COC, PP, and glass [58,59,60,61]. Tape bonding enables the microchannel fabrication at room temperature with single- or double-sided adhesive tape. The double-sided tape can replace the etching process of glasses, because channels can be patterned directly onto the tape [62]. However, the technique seems to have difficulty fabricating complex profiles of microchannels with multiple levels. The lateral surfaces of microchannels retained the weak tape material, which has low chemical resistance, affecting the reproducibility of experiments [63].

In this work, we propose an epoxy-based polymeric microfluidic device using a simple soft lithography process. Our proposed epoxy-based microfluidic device not only provides a simple manufacturing process, but also enables mass production and large-area fabrication of microfluidic devices. Thus, we have successfully fabricated microfluidic devices from slide glass-sized microchannel replicas for general applications to 6-inch wafer-sized replicas, which are difficult to fabricate at a laboratory level. The manufacturing processes we have developed, including reusable PDMS master molds and wafer-scale replicas, demonstrate the potential for high throughput production in the laboratory. In this study, we introduced the fabrication process of polymeric microfluidic devices with various dimensions, and performed physical and chemical resistance tests by comparing with conventional PDMS channels. First, we fabricated polymeric microfluidic devices based on soft PDMS molds, using combination of conventional photolithography and soft lithography. Second, in order to demonstrate the robustness of the polymeric devices, we measured the change in flow velocity inside the channel using fluorescent particles at various flow rate conditions. Third, we compared the diffusion of fluorescent particles over time in both microchannels in order to visualize chemical absorption. Finally, in order to compare suitability for day-long experiments, the pervaporation in compartmentalized microchambers was observed for 24 h, based on the Brownian motion of fluorescent particles. Through a series of experiments, we demonstrated the suitability of polymeric devices for robust and day-long experiments. We believe that epoxy-based polymeric devices can be used to facilitate microfluidic experiments when PDMS devices are not suitable.

## 2. Experimental Section

### 2.1. Reagents and Materials

The polymeric microfluidic devices used in the experiments were fabricated using epoxy resin (EpoxAcast 690, Smooth-on, Macungie, PA, USA). PDMS (Sylgard 184, K1solution, Gyeonggi-do, Korea) was used to fabricate microfluidic devices for comparison with epoxy-based polymeric devices. Yellow-green fluorescent polystyrene particles (latex beads, Sigma–Aldrich, Seoul, Korea) with a diameter of 1 µm were diluted with distilled ratio at a ratio of 1:1000; they were then used to visualize the flow and observe water evaporation in the compartmentalized microchambers. *Rhodamine B* (Sigma–Aldrich) was diluted to 10 µM in distilled water and used to compare the molecule absorption of the polymer and PDMS surfaces. Fluorocarbon oil (Fluorinert FC-40, Sigma–Aldrich) was used to compartmentalize the microchambers.

### 2.2. Device Design and Fabrication

The PDMS microfluidic device was fabricated and prepared using standard soft lithography, based on our previous study [64,65]. Briefly, a negative photoresist (SU-8 2025 and 2100, MicroChem, Newton, MA, USA) was used to fabricate the master mold through standard photolithography processes. The master mold was coated with *trichloro(3,3,3-trifluoropropyl)silane* (Sigma–Aldrich) in a vacuum jar for 1 h. Subsequently, PDMS solution was degassed in a vacuum jar, cast, and cured at 60 °C for 4 h. The PDMS channel and slide glass were directly bonded via oxygen plasma treatment (Cute-MP, Femto Science, Gyeonggi-do, Korea) at 50 sccm of O_2_ and 50 W for 30 s. After integration, the microfluidic channels were coated with 0.01% pluronic surfactant (F-127, Sigma–Aldrich) to minimize the non-specific binding of molecules. In order to prevent contamination, every microfluidic device used in this study was used only once per experiment.

### 2.3. Fabrication and Preparation of Polymeric Microfluidic Chips

**Figure 1:**
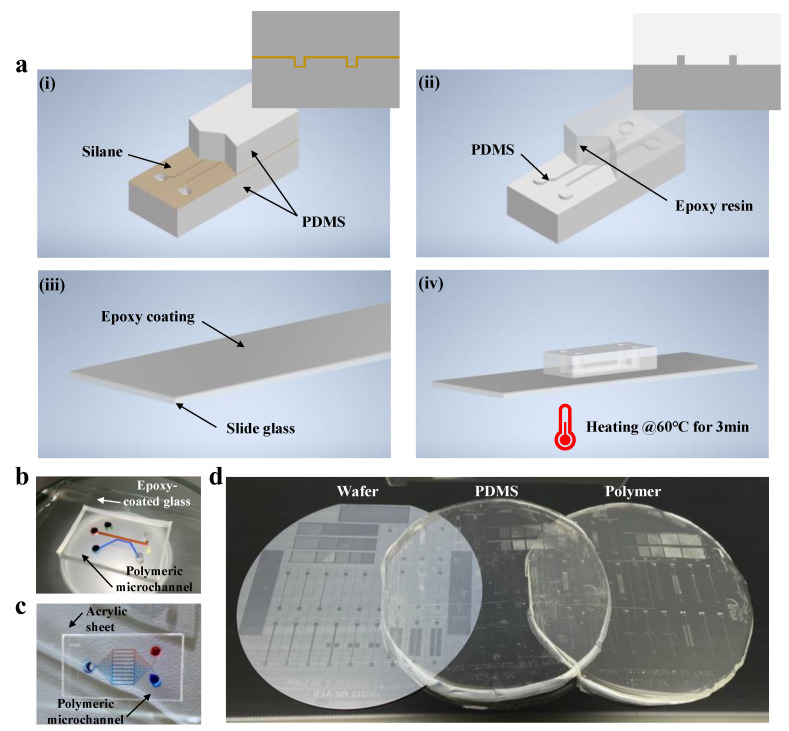
Illustration and photographs of fabrication process of epoxy-based microfluidic devices: (**a**) fabrication process of the polymeric devices. (i) Silane was coated on lower PDMS piece. PDMS was poured onto the lower piece to create a master mold for the polymeric devices, and cured at 60 °C for 2 h. (ii) The upper piece of (i) was peeled off and flipped over. Epoxy resin was poured onto PDMS and cured for 24 h at room temperature. (iii) Epoxy resin was poured onto a piece of glass and cured for 24 h at room temperature. (iv) Patterned epoxy was placed on the epoxy-coated glass and baked at 60 °C for 3 min on a hot plate. (**b**) Photograph of polymer-based microfluidic chip filled with dye. The device was fabricated via the abovementioned process. (**c**) Photograph of polymer device integrated with acrylic sheet. (**d**) From left to right: 6-inch wafer, PDMS, and polymer. For PDMS and polymer, wafer and PDMS were used as master molds, respectively.

In this study, we developed rigid and easily fabricated polymer-based microfluidic devices using epoxy resin. The fabrication process of the polymeric device is shown in Figure 1a. The surface of PDMS was silanized at 60 °C for 2 h, and PDMS was poured on the silane-coated PDMS channel. Although the PDMS channel and solution are homogeneous materials, they can separate easily, owing to the nanoscopic layer between them. Next, the prepolymer of epoxy resin was mixed with the curing agent in a 10:3 (*w*/*w*) ratio and degassed in a vacuum jar. The degassed epoxy resin was poured on separated PDMS and cured for 24 h under room-temperature conditions. Furthermore, to fabricate epoxy-coated glass, epoxy resin was poured on a slide glass, spin coated at 2000 rpm for 30 s, and cured at the same condition. Finally, a polymeric microfluidic device was fabricated via heat treatment of a polymeric microfluidic channel and an epoxy-coated glass at 60 °C for 3 min on a hot plate. Similarly to the PDMS devices, the polymeric devices were coated with pluronic surfactant under the same conditions. The polymeric devices were used only once per experiment in order to prevent contamination. Figure 1b shows the polymeric microfluidic devices fabricated by the protocol. In addition, Figure 1c shows that polymeric microfluidic devices can be fabricated using not only epoxy-coated glass, but also commercially available acrylic sheets. Notably, an all-polymer-based microfluidic device was fabricated by bonding the polymer replica with acrylic sheets via heat treatment at 60 °C for 3 min on a hot plate. We also demonstrated the feasibility of fabricating polymer-based devices of various sizes using this protocol by fabricating wafer-sized batch fabrications (Figure 1d).

Regarding the fabrication process, it is worth discussing that our fabrication process still requires a cleanroom process and PDMS soft lithography. The three-step process of photolithography, PDMS master mold fabrication, and polymeric device fabrication can seem complicated. However, repeated photolithography and soft lithography are not necessary to repeatedly produce the polymer chips, and can be omitted once the PDMS master mold is prepared, as it is reliable and reusable.

### 2.4. Experimental Setup and Data Analysis

A digital fluorescence microscope (F1-CIS, Nanoscope Systems, Daejeon, Korea), equipped with a charge-coupled device camera and lens, was used to acquire bright-field, green fluorescent, and red fluorescent images. Syringe-pump-driven flow was generated to control the flow rate in the microfluidic channels (NE-1000, New Era Pump Systems, Inc., Farmingdale, NY, USA). The ImageJ software (NIH, Bethesda, MD, USA) was used to measure the microchannel size, fluorescence intensity, and particle displacement.

## 3. Results and Discussions

### 3.1. Dimension Comparison of Polymer and PDMS Devices

**Figure 2:**
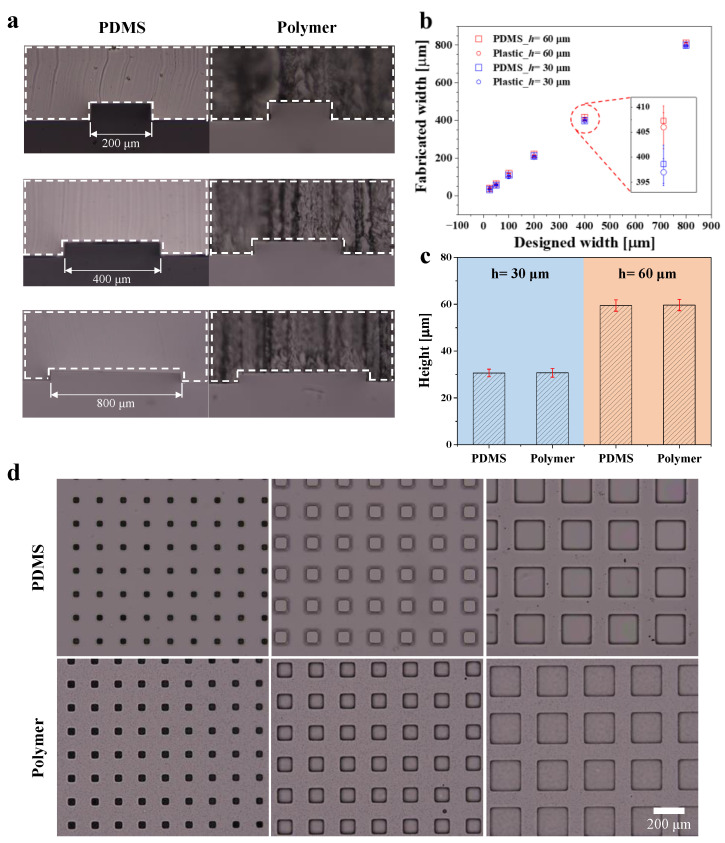
Dimension comparison of polymer and PDMS devices. (**a**) Bright-field images of cross-sections of PDMS channels (left) and polymer channels (right). Channel widths were 200, 400, and 800 μm from the top, and height was 60 μm. (**b**, **c**) Comparison of (**b**) width and (**c**) height in each channel. (**d**) Bright-field images of the square-shaped microchamber array. Scale bar represents 200 μm.

As shown in Table 2, the epoxy resin has better mechanical properties and lower gas permeability than PDMS [13,66]. Another advantage of epoxy resin is that shrinkage during curing is negligible (~0.2%) [50,67,68]; therefore, the fabricated devices will exhibit dimensions similar to those of the PDMS master mold. Here, we measured the channel dimensions of the PDMS master mold and the polymeric microfluidic channel. First, we obtained high-resolution optical images of the microchannels using a microscope at 10× magnification, and then measured the size of the micropatterns based on the number of pixels. Because the size corresponding to single pixel is about 0.55 μm, we considered that the error on the indirect measurement of the size is less than the pixel resolution, which seems to be negligible. Figure 2a shows the cross-section of the PDMS and polymer microchannel in variously designed channel widths. In order to measure the dimensions, the cross-sections of the PDMS master mold (left) and polymer microchannel (right) were cut and marked with white dashed lines. Unlike the images of PDMS, those of the polymer microchannel were opaque and rough, because a saw machine was used to cut the cross-section. By contrast, the PDMS cross-section shows relatively smooth surfaces, because a razor blade was used for cutting the PDMS channel. However, we confirmed through microscopic imaging that the difference in roughness of the cutting surfaces was due to different cutting methods, and that the transparency of the polymeric microfluidic device was comparable to that of PDMS.

We measured the dimensions of both channels with widths of 25, 50, 100, 200, 400, and 800 μm, which were fabricated using master molds of two heights, i.e., 30 and 60 μm. Then, we compared the widths of the PDMS channels and the polymer microchannels fabricated with a PDMS master mold (Figure 2b). Subsequently, we calculated the average dimensions by measuring five samples, for both the PDMS and polymer channels, via simple image processing. The red dash in Figure 2b shows that the dimensional error caused by the shrinkage of the epoxy resin was sufficiently low (<0.5%). In addition, the heights of the microchannels were measured and compared in the same manner (Figure 2c). The measurement results of the channels with heights of 30 μm (blue in Figure 2c) and 60 μm (orange in Figure 2c) similarly indicate that the fabrication error was negligible. Thus, we demonstrated that the shrinkage of the epoxy resin did not significantly affect the fabrication of polymeric microfluidic devices using a PDMS-based soft mold. In addition, because the dimensions of the polymer channel were almost identical to those of the PDMS channel, we fabricated channels for a square-shaped microchamber array in order to demonstrate that channels of various shapes can be fabricated (Figure 2d). The upper and lower channels shown in the figure were constructed using PDMS and epoxy resin, respectively. The lengths of one side of the square were 50, 100, and 200 μm from the left.

### 3.2. Channel Deformation at Various Flow Rates

**Figure 3:**
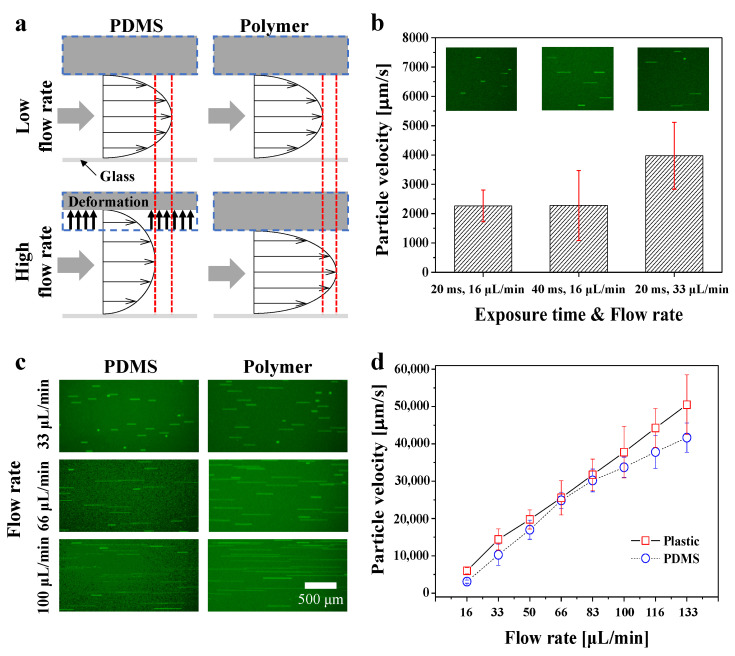
Channel deformation due to pressure-driven flow. (**a**) Schematic illustration of deformation of PDMS channel due to pressure-driven flow. Dashed blue and red lines represent channel deformation with pressure and decrease in velocity profile with deformation, respectively. (**b**) Particle velocity measurement in PDMS channels at different exposure times and flow rates. Inset images show displacement of particles at each condition. (**c**) Fluorescence images showing displacement of particles in each channel at flow rates of 33, 66, and 100 μL/min. Scale bar represents 500 *μ*m. (**d**) Particle velocity measured based on flow rate in each channel. At low flow rates, the particle velocity in the PDMS channels was smaller than that in the polymer channels.

Figure 3a shows a schematic illustration of the deformation of the PDMS microchannel under flows at both high and low flow rates. The high flow rate causes deformation of the PDMS channel, producing a smoother velocity profile compared to polymer channels at the same flow rate. On the other hand, since the polymeric device is not deformed, the velocity profile becomes steeper with the increasing flow rate. In addition, PDMS microchannels can deform even at a relatively low flow rate (15 nL/min) [69]. The theoretical flow rate at the rectangular microchannel with a no-slip boundary condition is expressed by Equation (1) [70].

(1) $$\begin{array}{ll} Q & =2\int_{0}^{\frac{1}{2}w}\int_{0}^{h}u_{x}dydz\\ & =\frac{wh^{3}\Delta p}{12\mu L}\left[1-\sum_{n=1,3,5\cdots }^{\infty }\frac{1}{n^{5}}\frac{192}{\pi^{5}}\frac{h}{w}\tanh(n\pi \frac{w}{2h})\right] \end{array}$$

where *w*, *h*, and *L* are the width (*y* direction), height (*z* direction), and length (*x* direction) of the microchannel, respectively, Δ*p* is the pressure drop, and *µ* is viscosity of the solution. However, due to PDMS bulging, flow velocity is difficult to predict, because the theoretical and experimental values are normally different in the PDMS microchannel. In particular, in experiments that require precise control of the flow rate, the use of PDMS may not be suitable. The desired flow rate at the PDMS channel can be calculated via numerical simulation by solving the fluid–solid interaction problem; however, various variables (e.g., wall thickness, elastic modulus, etc.) may be different from case to case [71]. However, because the elastic modulus of polymeric microfluidic devices fabricated using epoxy resin is extremely high compared with that of PDMS, the deformation due to syringe-pump-driven flow is negligible at typically applied pressure ranges in microfluidics, showing flow behavior in a rigid channel.

In order to demonstrate the robustness of the polymeric devices, we indirectly measured the deformation of both devices by tracking yellow-green fluorescent particles at various flow rates. Figure 3b shows the velocity calculation data obtained using fluorescent particles in the PDMS microchannel. The flow velocity of the channel was measured based on the fluorescent particle trajectory (streakline) by controlling the flow rate of the syringe pump and the exposure time of the camera. The inset images in the figure are fluorescence images showing the streaklines at different flow rates and camera-exposure times. We calculated the particle velocity based on the exposure time of the camera at flow rates of 16 and 33 μL/min in order to ensure the reliability of the measurement. First, at an exposure time and flow rate of 20 ms and 16 μL/min, respectively, the particle velocity was equal to that at 40 ms and 16 μL/min. At a flow rate of 33 μL/min, the particle velocity almost doubled compared with that of the previous two conditions. Next, in order to demonstrate the pressure resistance of the polymer channels, we measured the particle velocity in both channels with exposure times of 20 ms from 16 to 133 μL/min.

Figure 3c shows the streakline of particles at flow rates of 33, 66, and 100 μL/min in each channel. After obtaining fluorescence images, the lengths of all streaklines were measured and the flow velocity was calculated using the top 30% length of particles passing through the center of the channel, assuming fully developed flow. Figure 3d shows the velocity of the fluorescent particles based on a flow rate from 16 to 133 μL/min in the PDMS (blue) and polymer channels (red), using the calculation method described above. The linear increase in the particle velocity with the flow rate at the polymer channels indicates that the deformation of the polymer is negligible. However, in the PDMS microchannel, the flow rate and particle velocity were shown to be non-linear, owing to pressure-induced deformation of the PDMS channel. In the graph, the error bars of the polymer device appear higher than those of the PDMS microchannel, because the velocity profile of the polymer device is steeper than the PDMS channel at the same flow rate, as described in Figure 3a. In addition to the linearity of the pressure and velocity, the particle velocity in the PDMS channels was lower than that in the polymer channels, including at low flow rates.

Furthermore, we measured the maximum pressure that each device could withstand using a home-built pneumatic-based microflow controller (Figure S1a). As shown in Figure S1b, leakage occurred after 91.6 and 138.3 μL/min in the PDMS and the polymeric device, respectively. In addition, while the flow rate of the polymer device increases linearly, the PDMS device has a larger increase than the polymer device, at a pressure of 0.4 MPa, due to the deformation by pressure. Figure S1c shows the PDMS channel before and after the leakage, according to the burst of the PDMS device. On the other hand, the leakage occurred at the junction rather than inside the polymeric device (Figure S1d). Although we did not measure the maximum pressure the polymer device could withstand due to leakage at the junction, we expect that the polymeric device could withstand higher pressure with firmly fixed tubing. In addition, 138.3 μL/min is a sufficient level of pressure, which can cover most of the microfluidic applications requiring pressure-driven flow, such as particle separation, drug delivery, and microflow stabilizer.

### 3.3. Visualization and Quantification of Molecular Adsorption

**Figure 4:**
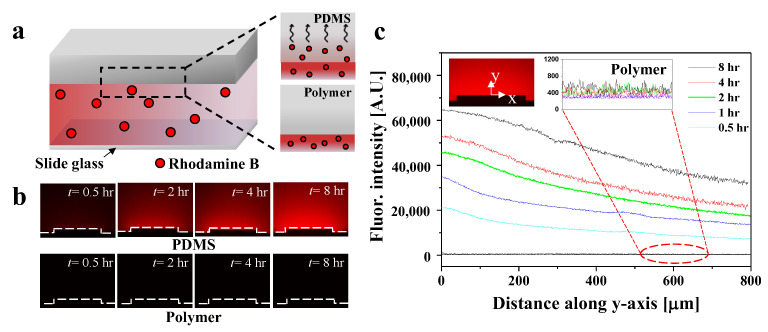
(**a**) Schematic illustration of diffusion of *Rhodamine B* molecules expressing red fluorescence. Dashed black line shows the adsorption of molecules on the surface of each channel. (**b**) Fluorescence images showing diffusion of *Rhodamine B* over time. Dashed white line represents the surface of the channels. (**c**) Quantification of fluorescence intensity in each channel. As shown in inset image, fluorescence intensity was measured along the *y*-axis with respect to the center of the channel. Dashed red line shows measurement of the fluorescence intensity in polymer channels.

We measured the fluorescence intensity in order to compare the molecular absorption of PDMS and polymer channels using hydrophobic fluorescence molecules. Figure 4a illustrates the experimental procedure using fluorescent *Rhodamine B* in both channels. First, we filled the channels with *Rhodamine B* solution, then stored them at room temperature to allow sufficient diffusion of the solution. Additionally, we minimized light irradiation to prevent the degradation of the fluorescence intensity. The dashed black line in the figure shows the diffusion of *Rhodamine B* molecules through the channel wall. In the PDMS channels, the fluorescent molecules were absorbed owing to the permeable characteristics of PDMS; however, in the polymer channels, the fluorescent molecules were not detected. We observed the cross-section of each channel using a digital fluorescence microscope every 0.5, 1, 2, 4, and 8 h. Figure 4b shows the visualization of the absorption of *Rhodamine B* over time. In the PDMS channels, the diffusion of fluorescence with time occurred along the wall of the channel, as indicated by the dashed white line. Compared with the polymer channels, the PDMS channels showed increased fluorescence intensity over time, owing to molecule absorption of PDMS. The fluorescence intensity was quantitatively measured based on the diffusion of *Rhodamine B*, and the results are shown in Figure 4c. The fluorescence intensity was measured along the *y*-axis from the center of the upper surface of the channel, as shown in the inset. In the PDMS channels, the fluorescence intensity increased over time. At 0.5 and 8 h, the fluorescence intensity increased by almost three-fold. As shown by the dashed red line, the fluorescence intensity in the polymer channels did not change with time.

### 3.4. Particle Compartmentalization for Day-Long Experiments

**Figure 5:**
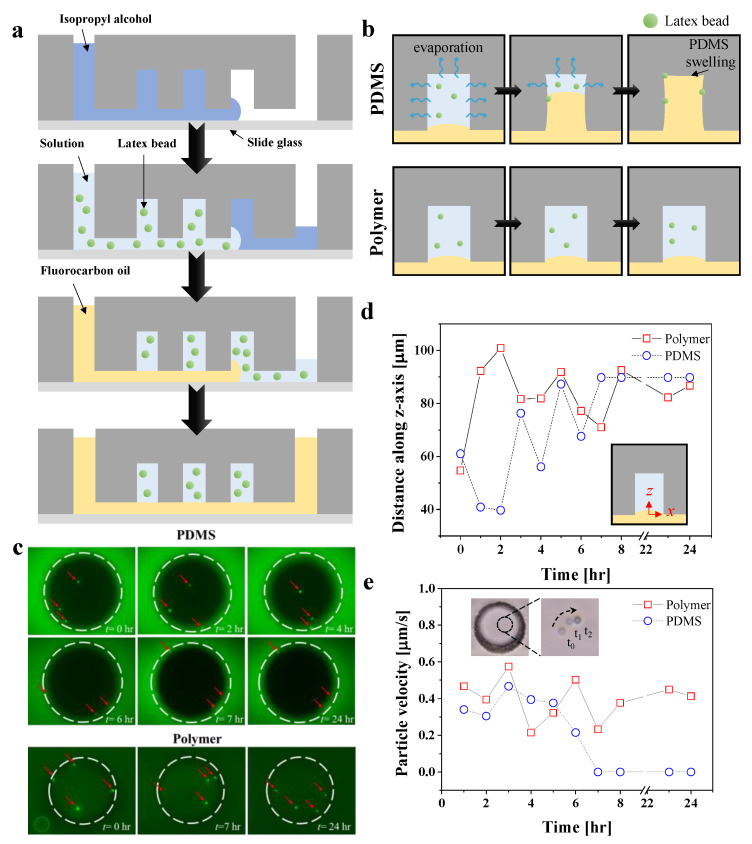
(**a**) Schematic illustration of particle compartmentalization. In order to visualize the evaporation of microchambers, latex beads expressing green fluorescence were diluted in distilled water. (**b**) Schematic illustration of the evaporation process inside microchambers in each channel. (**c**) Fluorescence images of microchambers containing fluorescent particles in each channel. Red arrows indicate fluorescent particles with a diameter of 1 μm. Dashed white circles represent microchambers with a diameter and height of 100 μm. (**d**) Vertical motion of fluorescent particles was quantified as a position along the *z*-axis. (**e**) Planar motion of the fluorescent particles was quantified as particle velocity. Particle displacement was measured for 5 s. Inset images show Brownian motion of particles in microchamber.

Next, we observed evaporation in the channels in order to compare their suitability for day-long experiments. The evaluation was performed via particle compartmentalization, using yellow-green fluorescent particles with a diameter of 1 μm. Figure 5a shows the device preparation procedure for the particle compartmentalization. First, *isopropyl alcohol* (IPA) was loaded to flush air, in order to prevent it from being trapped in the dead-end chambers. Due to its amphiphilic property and lower contact angle [72], IPA reduces the formation of air bubbles in the dead-end chambers. Second, distilled water is flushed in the channel over 10 min in order to fully remove the IPA residuals trapped in the microchambers. The particle solution was then filled with an appropriate volume (>1 mL) in order to ensure that the particles were sufficiently supplied inside the microchambers. Finally, fluorocarbon oil, which is denser than water, was loaded in order to form compartmentalized chambers filled with an aqueous solution of fluorescent particles.

Figure 5b shows the evaporation process in the microchambers of the PDMS and polymer channels. We observed evaporation inside the chambers based on the *Brownian* motion and the locations of the fluorescent particles at room temperature. In the PDMS channels, the solution inside the chambers evaporated over time due to air permeability (pervaporation), causing the particles to adhere to the wall and halt *Brownian* motion. Additionally, absorption of oil molecules also occurred, leading to swelling of the microchamber [73]. Meanwhile, the particles in the polymer-based microchambers showed continued movement over 24 h. We visualized the *Brownian* motion of the fluorescent particles every 1 h in each channel. The evaporation of the solution inside the chambers was monitored by using a digital fluorescence microscope (Figure 5c). In the video showing movement of fluorescence particles in the PDMS channels, the particles exhibited *Brownian* motion for up to 6 h; however, after 7 h, they stopped the motion because the solution inside the chambers evaporated, and the particles were stuck to the chamber wall. Compared with the particles in the PDMS channels, those in the polymer channels exhibited continuous and random *Brownian* motion over the day-long experimental process.

A quantification of the vertical and planar motions of the fluorescent particles is shown in Figure 5d,e, respectively. First, in order to measure the vertical motion, a particle located at the top of the particles inside the chamber was selected. The position of the particle was measured from the bottom of the chamber as a reference point (see schematic illustration in Figure 5d). In the polymer channels, the particle motion along the *z*-axis changed over time, whereas in the PDMS channels, the particles stopped moving after 7 h. Next, the planar motion of the particle was quantified as a velocity by measuring the displacement of the particle for 5 s. The inset images in Figure 5e show the particle motion over time. Similar to the results for vertical motion, the particles in the PDMS channels stopped moving after 7 h, whereas the particles exhibited *Brownian* motion continuously in the polymer chambers. It would be worthwhile to discuss that the solvent evaporation time in a nano- or picoliter-sized microchamber is generally less than an hour [74]. Although the evaporation rate of the solvent may depend on the thickness of the PDMS and other environmental conditions, such as humidity and temperature, our device seems to have the advantage of keeping the tiny droplet until most chemical reactions or bioassays are completed. In this context, we believe that the 24 h experiment we have demonstrated can be further extended according to the needs of the user, and is an experimental time scale which is practically long enough.

## 4. Conclusions

Herein, we present an inexpensive and easy-to-fabricate polymeric microfluidic device using epoxy resin that can overcome the inherent disadvantages of PDMS, such as elastic deformation due to pressure, molecule absorption, and air permeability. The single process time of 24 h is relatively longer than PDMS devices, but we demonstrated that the fabrication process of proposed polymer devices allows for massive and parallel fabrication, using multiple PDMS replicas as master molds that can be easily prepared using conventional soft lithography. Thus, fabrication time can be compensated for by simultaneously producing multiple polymer devices in a parallel manner. Next, we measured the dimensions of the epoxy-based polymer channels fabricated using PDMS channels as a master mold, and we confirmed that the dimensional error (<0.5%) of the fabrication process was negligible. Additionally, we demonstrated that epoxy resin can be used to fabricate various types of designs through the fabrication of 6-inch wafer replicas and square-shaped microchamber arrays. In order to compare the rigidity of each device, we successfully conducted an experiment to measure the velocity change of the particles based on the flow rate (~133 μL/min) in the PDMS and polymeric devices, the results of which confirmed that the polymer channels exhibited negligible deformation in typical flow rate ranges in microfluidics. By contrast, deformation of the PDMS channel was observed, including a relatively low flow rate (16 μL/min) generated by a syringe pump. Another advantage of polymeric microfluidic devices is that they barely absorb water, oil, or small hydrophobic molecules. We demonstrated the excellent chemical resistance of the polymer devices by measuring fluorescence intensity over time in each channel, using a *Rhodamine B* solution. In addition, the suitability of the polymer devices for day-long experiments was confirmed by comparing the evaporation inside the microchambers through particle compartmentalization. Based on a series of demonstrations, we confirmed that the polymeric microfluidic devices were more robust and suitable for chemically demanding and day-long experiments than the PDMS microfluidic devices. In addition, since the proposed fabrication process enables massive and parallel fabrication at a low cost, we have confirmed its potential for commercialization for various analytical fields which require automation and mass production. Hence, we believe that the proposed epoxy-based polymeric microchip will facilitate not only fundamental experiments involving microfluidics, but also biological applications, such as high-performance liquid chromatography, anaerobic bacterial culture, and polymerase chain reaction.

## Figures and Tables

**Table 1:** Physical properties of polymer for microchannel fabrication.

	PDMS	COC	PP	Epoxy
Young’s modulus (MPa)	2.05 ± 0.12	2.60 × 10^3^	0.77 × 10^3^	3.94 × 10^3^
Cure shrinkage	0.5–2.5%	0.1–0.5 %	1.7–2.2%	0.2%
Transparency	Good	Good	Medium	Good
Solvent resistance	Low (organic solvent)	Medium (non-polar organic solvent)	High	High
Reference	[18,20]	[35,37,38,48]	[40,41,42,49]	[45,50,51]

**Table 2:** Mechanical properties of PDMS and epoxy resin.

	Elongation Limits (mm)	Young’s Modulus (MPa)	Ultimate Tensile Strength (MPa)	Compressive Modulus (MPa)	Compressive Strength (MPa)	Gas Permeability
PDMS [66]	76.4	2.05 ± 0.12	6.25 ± 0.84	148.9 ± 5.47	40.1 ± 4.30	Good
Epoxy resin [50]	1.8	3.94 × 10^3^	45.7	6.30 × 10^2^	66.3	Low

## Data Availability

Not applicable.

## Supplementary Materials

The following supporting information can be downloaded at: https://www.mdpi.com/article/10.3390/bios12100838/s1, Figure S1: Testing the maximum pressure that the PDMS or polymer microfluidic devices can withstand.
