# Supplementary material for: Polymeric Microfluidic Devices Fabricated Using Epoxy Resin for Chemically Demanding and Day-Long Experiments
Source: Biosensors (Basel). 2022 Oct 7;12(10):838. doi: 10.3390/bios12100838 (PMC9599855; doi:10.3390/bios12100838)
Supplement: Supplementary file 1 [file biosensors-12-00838-s001.zip › biosensors-1906064-supplementary.pdf]

biosensors

Supplementary Materials

Polymeric Microfluidic Devices Fabricated Using Epoxy Resin for Chemically Demanding and Day-Long Experiments

Jaeseok Lee [1,2] and Minseok Kim [1,2,*]

1 Department of Mechanical System Engineering, Kumoh National Institute of Technology, Gumi 39177, Korea
2 Department of Aeronautics, Mechanical and Electronic Convergence Engineering, Kumoh National Institute of Technology, Gumi 39177, Korea

Supplementary Figures

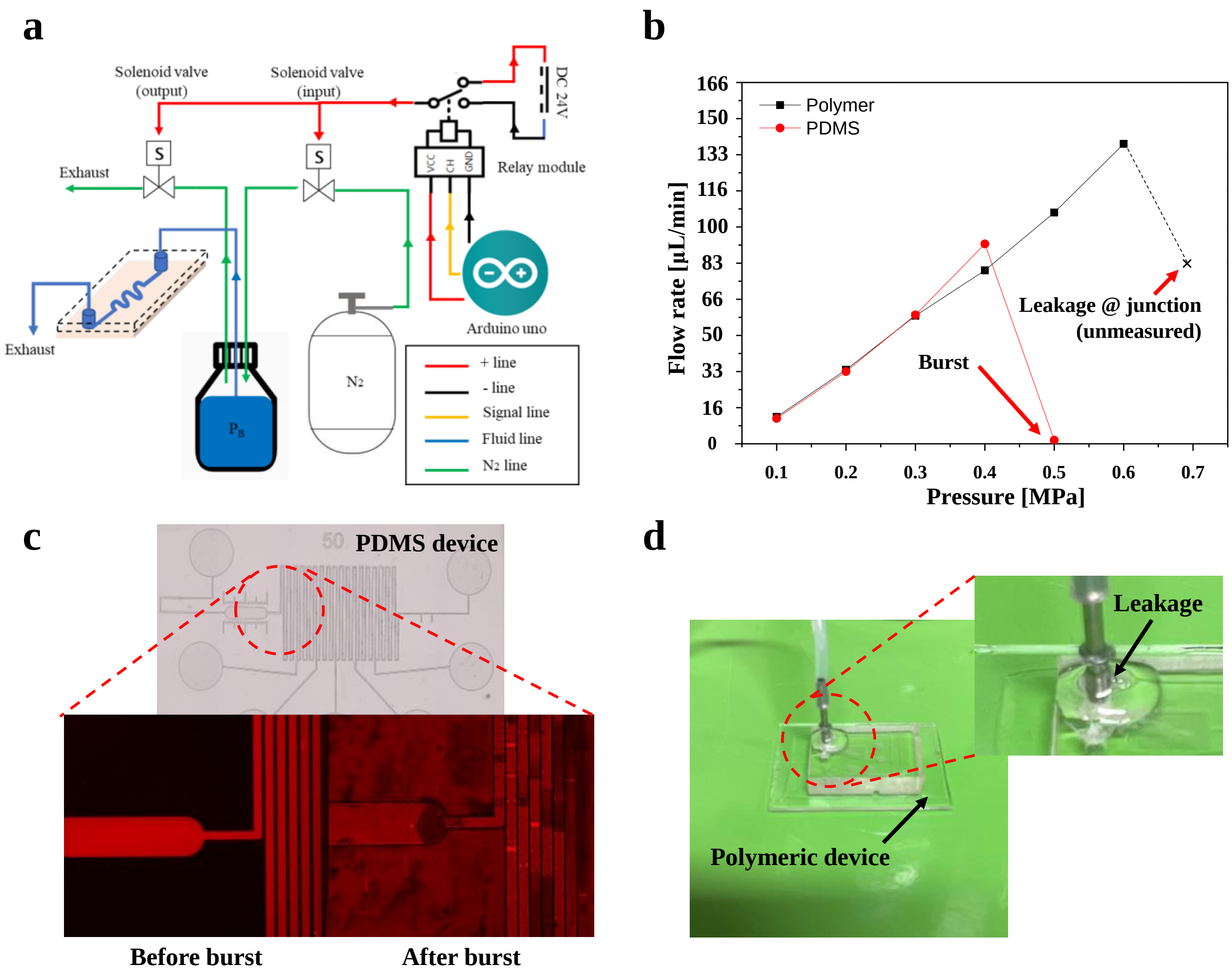

Figure S1. Testing the maximum pressure that the PDMS or polymer microfluidic devices can withstand. (**a**) The schematic illustration of home-built pressure-driven flow generator. (**b**) Change of flow rate according to pressure. In the PDMS microfluidic device, leakage occurs after 91.6 μL/min due to burst of microdevices. The leakage occurred at the junction after 138.3 μL/min at polymeric device. In addition, the flow rate of the polymeric device changes linearly, but in the PDMS device, the increase is larger at 0.4 MPa than the polymeric device due to the pressure-driven deformation. (**c**) Photograph of PDMS channel before and after the leakage, respectively. Leakage is caused by the separation of the PDMS from the slide glass by the high pressure. (**d**) Photograph of polymeric microfluidic device. The leakage occurred at the junction, not inside the polymeric device.
